# The Effects of Hydrogen Annealing on Carbon Nanotube Field-Effect Transistors

**DOI:** 10.3390/nano11102481

**Published:** 2021-09-23

**Authors:** Takashi Uchino, Greg N. Ayre, David C. Smith, John L. Hutchison, C. H. de Groot, Peter Ashburn

**Affiliations:** 1 Department of Electrical and Electronic Engineering, Tohoku Institute of Technology, Sendai 982-8577, Japan; 2 School of Physics and Astronomy, University of Southampton, Southampton SO17 1BJ, UK; g.n.ayre@phys.soton.ac.uk (G.N.A.); dcsmith@phys.soton.ac.uk (D.C.S.); 3 Department of Materials, University of Oxford, Parks Road, Oxford OX1 3PH, UK; john.hutchison@materials.ox.ac.uk; 4 School of Electronics and Computer Science, University of Southampton, Southampton SO17 1BJ, UK; chdg@ecs.soton.ac.uk (C.H.d.G.); pa@ecs.soton.ac.uk (P.A.)

**Keywords:** carbon nanotube, field-effect transistors, hydrogen annealing, electron affinity, bandgap

## Abstract

We have systematically investigated the effects of hydrogen annealing on Ni- and Al-contacted carbon nanotube field-effect transistors (CNTFETs), whose work functions have not been affected by hydrogen annealing. Measured results show that the electronic properties of single-walled carbon nanotubes are modified by hydrogen adsorption. The Ni-contacted CNTFETs, which initially showed metallic behavior, changed their p-FET behavior with a high on-current over 10 µA after hydrogen annealing. The on-current of the as-made p-FETs is much improved after hydrogen annealing. The Al-contacted CNTFETs, which initially showed metallic behavior, showed unipolar p-FET behavior after hydrogen annealing. We analyzed the energy band diagrams of the CNTFETs to explain experimental results, finding that the electron affinity and the bandgap of single-walled carbon nanotubes changed after hydrogen annealing. These results are consistent with previously reported ab initio calculations.

## 1. Introduction

Carbon nanotube field-effect transistors (CNTFETs) are being extensively studied for various applications, including microprocessors, high-frequency electronics, and display electronics [1,2,3,4]. An appropriate choice of source/drain metal electrodes must be made to achieve the best performance from CNTFETs. A Schottky barrier plays a crucial role in CNTFETs to inject carriers from metal into the carbon nanotube [5]. Javey et al. have shown that Pd is an appropriate choice for p-FETs [6] because it has a high work function of *Φ*_M_ = 5.1 eV and has the technological advantage of good wetting between the Pd and the carbon nanotube [7]. Zhang et al. have fabricated high-performance n-FETs using Sc contacts and HfO_2_ gate dielectrics, where Sc has a low work function of *Φ*_M_ = 3.3 eV [8].

Reduction in a single-walled carbon nanotube’s work function due to hydrogenation has been reported theoretically [9,10] and experimentally [11]. Ab initio calculations suggest that hydrogenation significantly influences the band structure of single-walled carbon nanotubes, reducing the electron affinity by around 0.6 eV and increasing the bandgap by about 0.2 eV [9]. The reduction in work function in hydrogen-passivated graphene nanoribbons [12] and the bandgap opening of graphene have also been reported in graphene FETs [13]. The extensive use of Pd as a source/drain contact compromises the experimental verification of these calculations in CNTFETs, since hydrogen has a strong effect on the work function of Pd. The hydrogen absorption in Pd has been shown to decrease work function by 0.4–0.7 eV experimentally [14,15], and similar but smaller effects have been reported for Ti [16].

Only a few experimental studies have reported the effects of hydrogen on CNTFETs; however, these results are contradictory [17,18,19]. As for the heterojunction between pristine and hydrogenated carbon nanotubes, Kim et al. reported that hydrogen annealing increased the bandgap of carbon nanotubes by 1.9–3.6 eV [17]. The authors explained that the C–H bond enhanced SP^3^ hybridization and opened the bandgap. However, these results are a more significant effect than the experimental results of CNTFETs [18]. The as-made p-FETs capped with the ZrO_2_ layer were converted to n-FETs after hydrogen annealing [19]. In this case, it is not clear whether the conversion is attributed to hydrogen because carbon nanotubes are blocked from the hydrogen atmosphere. The conversion has also been reported in the Al_2_O_3_-capped CNTFETs after thermal annealing in a vacuum due to doping [20]. The effect of hydrogen annealing on the CNTFETs, therefore, remains an open question.

This paper presents experimental results on the effect of hydrogen annealing on the current–voltage (*I–V*) characteristics of Ni- and Al-contacted CNTFETs. Ni has a high work function of 5.2 eV, similar to that for Pd, and Al has a lower work function of 4.3 eV. Previous studies reported [21,22] that the work function of Ni is unaffected by hydrogen annealing. Thermo-transport measurements of hydrogen in metals showed that the hydrogen concentration in Ni was smaller than one-hundredth of those in Pd and Ti. Thus, the effect of hydrogen adsorption is negligible. Electrical measurement results of Al-contacted devices showed that the work function of Al was also not affected by the hydrogen annealing around 400 °C [14,23], since the interaction between Al and hydrogen was energetically unstable [24].

The experimental results on the Ni-contacted CNTFETs indicate that as-made metallic behavior is converted into semiconducting behavior after hydrogen annealing. The measured results on Al-contacted CNTFETs show similar behavior. We successfully demonstrated that hydrogen annealing at 400 °C for 30 min improves the performance of p-type CNTFETs. The proposed band diagrams are consistent with the results of the previously reported ab initio calculations [9].

## 2. Experimental Procedure

A p+ Si substrate (0.005 Ω∙cm) was employed as a back gate. After a standard cleaning process, we formed a gate dielectric stack of SiO_2_/HfO_2_ (45/10 nm). The SiO_2_ layer was thermally grown and the HfO_2_ layer was deposited by atomic layer deposition (ALD). The carbon nanotubes used in this work were grown by thermal chemical vapor deposition (CVD) using a combination of Fe and Ge nanoparticles as previously described [25]. The nanoparticles were initially formed in a plasma SiO_2_ layer. The 30 nm-thick SiO_2_ layer was deposited on HfO_2_ by plasma-enhanced CVD (PECVD) and densified at 950 °C. The top PECVD-SiO_2_ layer was then implanted with 5 × 10^15^ cm^−2^, 20 keV Ge, and annealed in nitrogen at 600 °C for 40 min to create Ge nanoparticles [26]. The top PECVD-SiO_2_ layer was then removed using an HF vapor etch to expose the Ge nanoparticles on the HfO_2_ layer. Then the substrate was dipped in ferric nitrate solution for 1 min and rinsed with hexane. The CNT growth was carried out using CVD in a hot-wall reactor at atmospheric pressure. CNTs were grown at 850 °C for 20 min using a mixture of methane (1000 sccm) and hydrogen (300 sccm) immediately after a pre-anneal in hydrogen (1000 sccm) at 900 °C.

**Figure 1:**
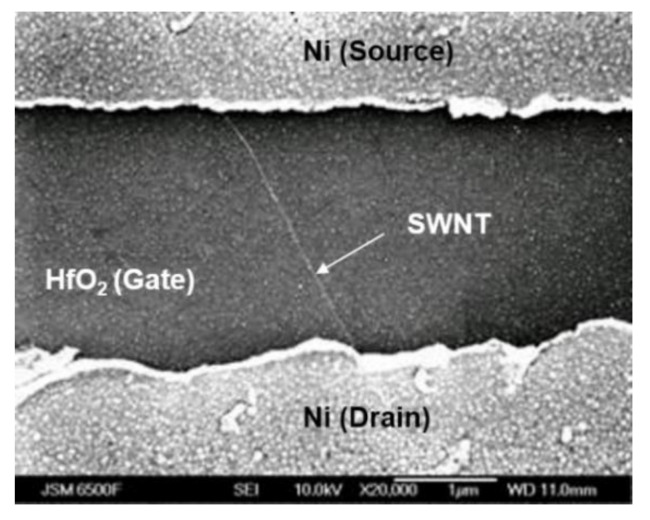
SEM image of the back-gate Ni-contacted CNTFET with SiO_2_/HfO_2_ gate dielectrics. An isolated single-walled carbon nanotube (SWNT) is presented.

Back-gate both Ni and Al-contacted CNTFETs were fabricated using the lift-off technique. The metals were deposited by sputtering, and the source/drain electrodes were formed using direct-write optical lithography and lift-off. The gap between the source/drain electrodes was 2 µm and the width was 5 µm, as shown in Figure 1. After source/drain formation, the devices were annealed in hydrogen (1000 sccm) at 400 °C for 30 min.

The synthesized carbon nanotubes were characterized using field emission scanning electron microscopy (FE-SEM, Hitachi, Tokyo, Japan), high-resolution transmission electron microscopy (HRTEM, JEOL, Tokyo, Japan), and Raman spectroscopy (Renishaw, Gloucestershire, UK). TEM sample preparation consisted of scraping the sample’s surface with a surgical blade and transferring it onto a carbon-coated Cu grid. Raman spectra were obtained using a micro-Raman system with He-Ne laser excitation (632.8 nm) at a power of 12 mW. *I–V* measurements were carried out before and after hydrogen annealing using an Agilent 4155C semiconductor parameter analyzer (Agilent Tech, Santa Clara, CA, USA). We used a Cascade REL3200 probe station (Cascade Microtech, Livermore, CA, USA) for the room temperature measurements and a Nagase BCT-43MDC probe station (Nagase, Tokyo, Japan) for the low-temperature measurements. The room temperature measurements were carried out in the air. The low-temperature measurements were carried out in a vacuum of 1.3 × 10^−4^ Pa after the samples were kept in a high vacuum for 30 h to exclude the effects of water and oxygen.

## 3. Results

Figure 1 shows an SEM image of the fabricated back-gate CNTFET with a SiO_2_/HfO_2_ gate dielectric and Ni source/drain contacts. The area density of carbon nanotubes is low, and almost all carbon nanotubes are isolated.

**Figure 2:**
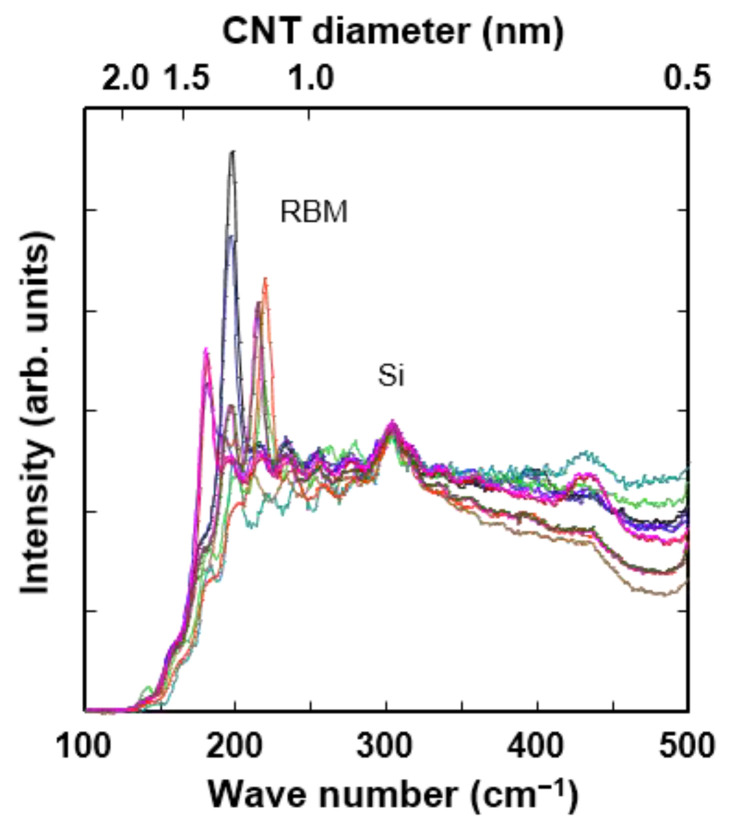
Raman spectra of carbon nanotubes grown from a combination of Ge and Fe nanoparticles on HfO_2_ substrate. Radial breathing mode (RBM) indicates that single-walled carbon nanotubes with diameters 1.2–1.8 nm are present.

Figure 2 shows the Raman spectra of the synthesized carbon nanotubes. All samples clearly show the radial breathing mode (RBM), suggesting that single-walled carbon nanotubes are present. The Raman intensity ratio of D-band to G-band is less than 0.1, indicating that synthesized single-walled carbon nanotubes have a low defect density. The diameter was estimated from the Raman spectroscopy results on a total of 18 measurements using the relation ω_RBM_ (cm^−1^) = 248/*d* (nm) [27]. The diameters of single-walled carbon nanotubes range from 1.2 to 1.8 nm, though diameters larger than 1.8 nm could not be observed due to the low-frequency cut-off of the Raman notch filter. The HRTEM results on 33 images show diameters ranging from 1.2 to 2.2 nm. This is in good agreement with the Raman estimate, given that the Raman notch filter prevents diameters larger than 1.8 nm from being observed. Thus, the HRTEM estimate of the maximum diameter of 2.2 nm is more reliable. The bandgap corresponding to semiconducting single-walled carbon nanotubes can be deduced from *E* = 0.9/*d* (nm) [28]. The bandgap ranges from 0.4 to 0.8 eV, and the mean bandgap is around 0.6 eV.

**Figure 3:**
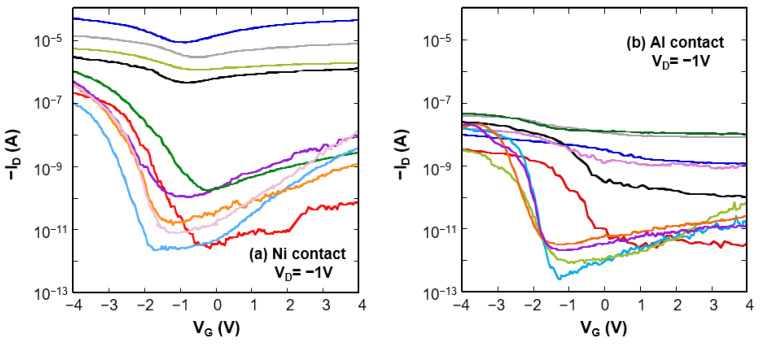
Sub-threshold characteristics of the CNTFETs after hydrogen annealing with (**a**) Ni-contacts and (**b**) Al-contacts.

*I–V* measurements on the CNTFETs gave more than 80 functional devices each for Ni- and Al-contacted devices. Figure 3 shows typical sub-threshold characteristics of the Ni- and Al-contacted CNTFETs after hydrogen annealing. The individual *I*–*V* characteristics are categorized into two main types: low-drain current with high *I*_ON_/*I*_OFF_ ratio and high-drain current with low *I*_ON_/*I*_OFF_ ratio. Hydrogen annealing has had a significant effect on the characteristics of the CNTFETs. To clarify the transformation behavior, we compare the *I*-*V* characteristics before and after hydrogen annealing.

**Figure 4:**
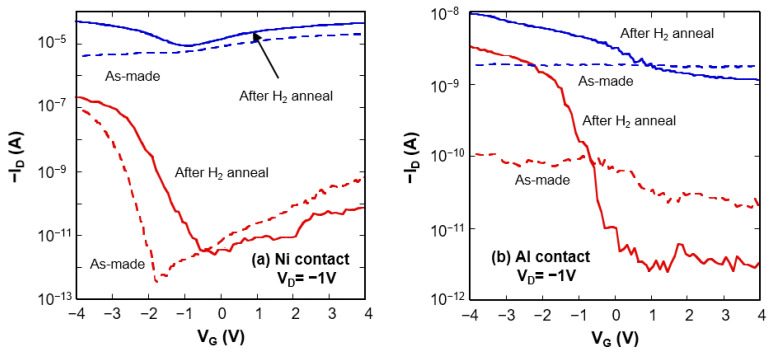
The transformation of sub-threshold characteristics of the CNTFETs with (**a**) Ni-contacts and (**b**) Al-contacts before and after hydrogen annealing.

Figure 4a shows sub-threshold characteristics for the Ni-contacted CNTFETs before and after hydrogen annealing. Hydrogen annealing improved the on-current of the CNTFETs. The as-made p-FET exhibited lower leakage current and higher on-current, comparable with Pd-contacted CNTFETs [29]. It is clear that a device initially showing metallic *I–V* behavior has gate voltage modulation of the drain current (gating effect) after hydrogen annealing, giving rise to the improved on-current. The top-gated graphene FETs have similar sub-threshold characteristics because of a zero or very narrow bandgap [30].

Figure 4b shows sub-threshold characteristics of the Al-contacted CNTFETs. The as-made devices show the metallic *I–V* characteristics with various current conductions that are associated with the bandgap variation of carbon nanotubes. The Al-contacted devices showed p-FET behavior after hydrogen annealing. Hydrogen annealing improved the on-current and leakage current. The device with higher current conduction turned into a p-FET with a lower *I*_ON_/*I*_OFF_ ratio. The large leakage current is attributed to the tunneling current across the narrow bandgap. The device with lower current conduction turned into p-FET behavior with the steep sub-threshold slope with a higher *I*_ON_/*I*_OFF_ ratio. The on-current of the Al-contacted CNTFETs is smaller than that of the Ni-contacted CNTFETs. As for Schottky barrier transistors, on-current is determined by the Schottky barrier height. Higher work function metals, such as Ni and Pd, form a lower Schottky barrier height; consequently, they induce higher on-current. Thus, these results suggest that the fabricated devices are relevant to Schottky barrier transistors.

**Figure 5:**
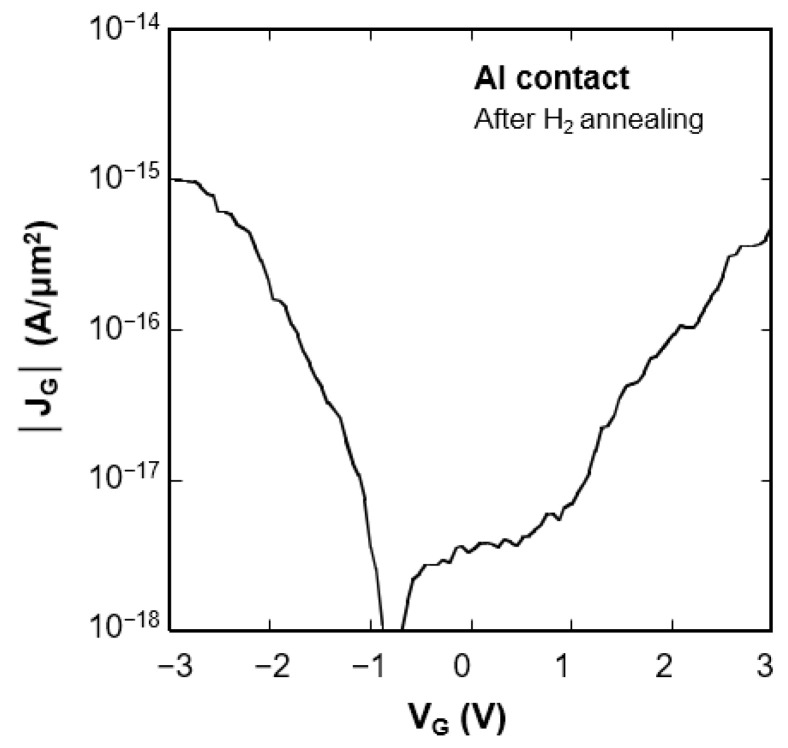
*J*_G_-*V*_G_ characteristics of the Al-contacted CNTFET after hydrogen annealing.

Figure 5 shows the gate voltage (*V*_G_) dependence of the gate leakage current density (*J*_G_) of the Al-contacted CNTFETs after hydrogen annealing. As the gate leakage current at 3 V is about three orders of magnitude smaller than the drain current of the CNTFET with a higher *I*_ON_/*I*_OFF_ ratio, the drain current is coming from the carbon nanotubes.

**Figure 6:**
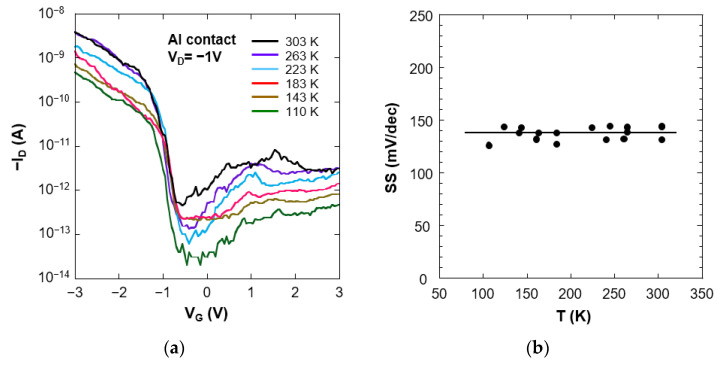
(**a**) Temperature dependence of sub-threshold characteristics of the Al-contacted CNTFETs after hydrogen annealing. Measurements were carried out at a drain voltage *V*_D_ = −1.0 V. (**b**) Temperature dependence of minimum sub-threshold slope *SS*.

The steep values of the sub-threshold slope of the Al-contacted CNTFETs suggest that Al/CNT contact has a lower Schottky barrier after hydrogen annealing. We measured the temperature dependence of the sub-threshold characteristics in a high vacuum to test this possibility. Figure 6a shows a decrease in drain current with decreasing temperature. Similar behavior has been reported on Pd-contacted CNTFETs [6] and was attributed to a lower Schottky barrier at source/drain contacts. The most likely explanation for forming the lower Schottky barrier at source/drain contacts is due to the modification of the electronic structure of carbon nanotubes. Hydrogen annealing decreases the electron affinity of carbon nanotubes and consequently leads to p-FET behavior. Figure 6b shows a temperature dependence of the sub-threshold slope, *SS* = (*d*log*I*_D_/*dV*_G_)^−1^. The sub-threshold slope is temperature independent and constant around 140 mV/dec. When the tunneling current through the Schottky barrier is dominant in drain current, the sub-threshold slope has temperature dependence. This is because the tunneling current has temperature dependence. The sub-threshold slope of temperature-independent and lower values, less than 200 mV/dec, indicate that the lower Schottky barrier height is formed at the contact region [31,32]. As for Schottky barrier FETs, the sub-threshold slope is sensitive to the electric field at the contact region; consequently, thinner gate dielectrics or higher dielectric constant is required to reduce the sub-threshold slope [33].

## 4. Discussion

**Figure 7:**
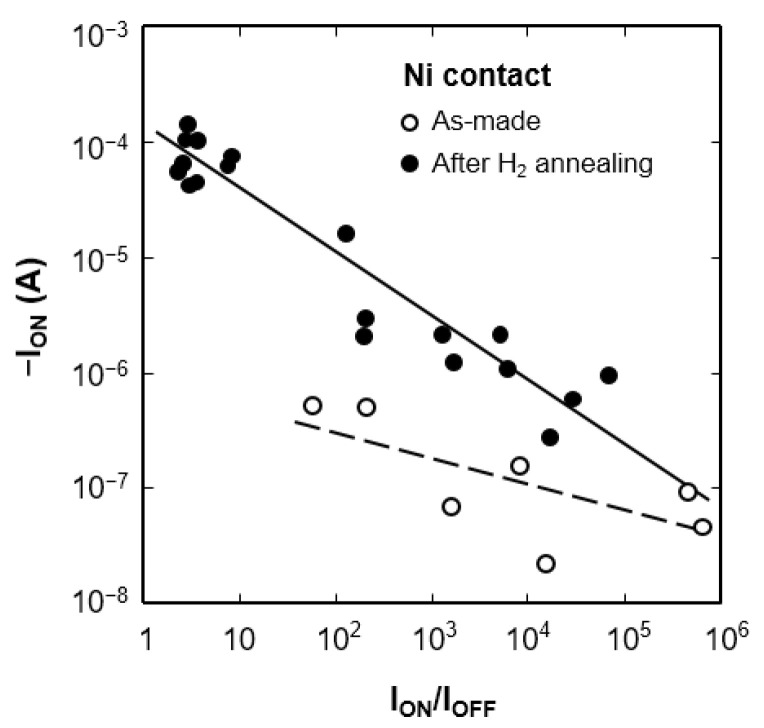
The correlation between *I*_ON_ and *I*_ON_/*I*_OFF_ ratio of the Ni-contacted CNTFETs.

Earlier research on Al-contacted CNTFETs has shown either p-type or n-type FET behavior [34,35,36]. These contradict results indicating that Al-contacted CNTFETs are easily influenced by ambient atmosphere. The absorbed oxygen can transform carbon nanotubes into p-type conductivity, and n-type conductivity can be formed to remove oxygen from carbon nanotubes surface [37,38]. To investigate the effect of oxygen, the hydrogen annealed Al-contacted CNTFETs were placed in a high vacuum of 1.3 × 10^−4^ Pa for 30 h (Figure 6). The CNTFETs still show p-FET behavior in a high vacuum. It is well known that hydrogen annealing effectively eliminates native oxide; therefore, the p-FET behavior is not a result of the absorption of oxygen. Thermal annealing in a vacuum improves on-current to reduce contact resistance [20]. However, it does not introduce the gating effect for the metallic carbon nanotubes. The most likely explanation for inducing the gating effect is the adsorption of hydrogen on carbon nanotubes, which could modify the electronic structure of carbon nanotubes due to the stable covalent C–H bonding [39]. Figure 7 summarizes the correlation between on-current (*I*_ON_) and *I*_ON_/*I*_OFF_ ratio before and after hydrogen annealing for the Ni-contacted CNTFETs. In all cases, hydrogen annealing leads to a significant increase in on-current, especially in the devices with an *I*_ON_/*I*_OFF_ ratio less than 100, which initially showed metallic behavior. The excellent correlation between on-current and *I*_ON_/*I*_OFF_ ratio indicates that the bandgap of carbon nanotubes determines on-current. The increase in on-current after hydrogen annealing could reduce the contact resistance and the reduced electron affinity of carbon nanotubes. Higher on-current over 10 µA is a result of the creation of a bandgap in carbon nanotubes. Narrow bandgap FETs exhibit high on-current due to increased mobility, and a small *I*_ON_/*I*_OFF_ ratio due to the large tunneling leakage current.

**Figure 8:**
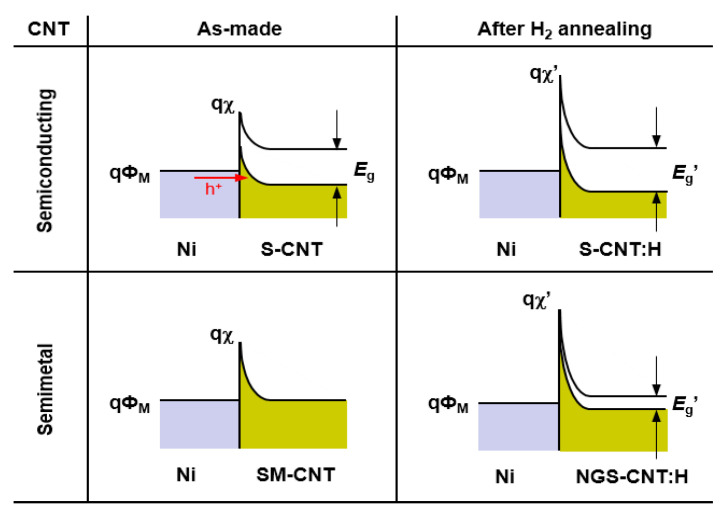
The proposed band diagrams for Ni-contacted CNTFETs. *E*_g_ and *χ* are bandgap and the electron affinity of a carbon nanotube, respectively, and *Φ*_M_ is the work function of Ni (5.2 eV).

To explain the effects of hydrogen annealing on the CNTFETs, the energy band diagrams of the Ni- and Al-contacted CNTFETs before and after hydrogen annealing are considered. It should be mentioned that the work functions of Ni and Al remain unaffected by hydrogen annealing, which differs from Pd. Figure 8 shows the energy band diagrams of the Ni-contacted CNTFETs, which are drawn based on the results of ab initio calculations. *E*_g_ and *χ* are bandgap and electron affinity of carbon nanotubes, and *Φ*_M_ is the work function of Ni. The estimated electron affinity and work function of the pristine carbon nanotubes were 4.2 and 4.5 eV, respectively [9]. These values are consistent with the previous results for single-walled carbon nanotubes [40]. The bandgap of carbon nanotubes increases by 0.2 eV, and the electron affinity is reduced by 0.6 eV after hydrogen annealing. The Schottky barrier for the hole was formed neither in the as-made nor after hydrogen annealing. In CNTFETs with the semimetal carbon nanotubes, creating a narrow bandgap delivers a lower *I*_ON_/*I*_OFF_ ratio and a higher on-current. As for the CNTFETs with semiconducting carbon nanotubes, the increase in bandgap improves on-current.

**Figure 9:**
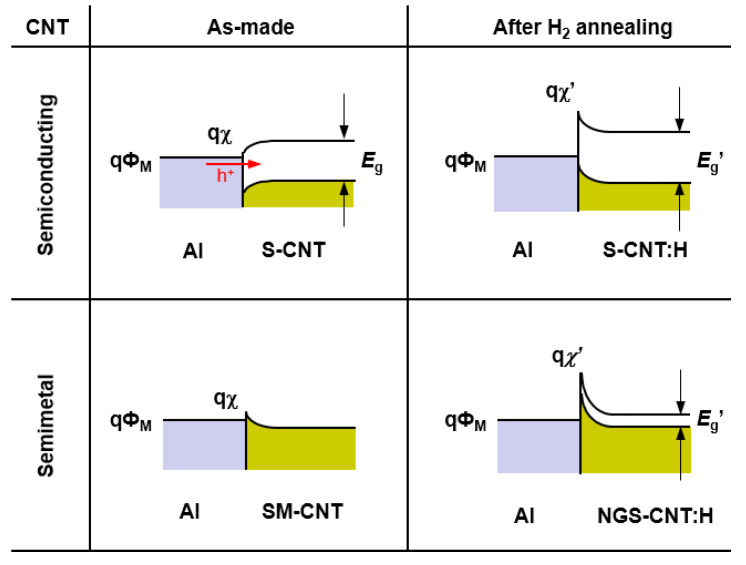
The proposed band diagrams for Al-contacted CNTFETs. *E*_g_ and *χ* are bandgap and the electron affinity of a carbon nanotube, respectively, and *Φ*_M_ is the work function of Al (4.3 eV).

Figure 9 shows the energy band diagrams of the Al-contacted CNTFETs. The band diagram for the as-made CNTFETs with semiconducting carbon nanotubes indicates that the lower work function of Al could produce n-FET behavior. Nonetheless, it is not stable because of the low Schottky barrier height. The CNTFETs with semimetal carbon nanotubes can convert from metallic to p-FET behavior after hydrogen annealing due to opening the bandgap and reducing the electron affinity of the carbon nanotubes. These results of the Al-contacted CNTFETs are consistent with the results of the Ni-contacted CNTFETs. In addition, the proposed model is supported by the ab initio calculations for hydrogenated carbon nanotubes.

As a final remark, we have also examined the effect of hydrogen annealing on CNTFETs with Pd source/drain contacts. However, we could not obtain any results because all the Pd films came off after hydrogen annealing; this was a result of hydrogen-induced plastic deformation.

## 5. Conclusions

In this paper, the effects of hydrogen annealing on CNTFETs have been systematically investigated using Ni- and Al-contacted CNTFETs. It emerges that the electronic characteristics of the CNTFETs are modified by hydrogen annealing, which can change the band structure of carbon nanotubes due to the presence of covalent C–H bonds. The Ni-contacted CNTFETs, which initially exhibited metallic behavior, were converted into p-FET behavior with a higher on-current and lower *I*_ON_/*I*_OFF_ ratio, due to creating a narrow bandgap in carbon nanotubes. The Al-contacted CNTFETs, which initially exhibited metallic behavior, were converted into unipolar p-FET behavior. We have introduced new energy band diagrams for the CNTFETs after hydrogen annealing. It is necessary to reduce electron affinity and widen the bandgap of carbon nanotubes to explain experimental results. These variations of parameters are supported by the ab initio calculations.

## Data Availability

The data presented in this study are available on request from the corresponding author.
